# Could Skin Autofluorescence Be a Useful Biomarker in Systemic Lupus Erythematosus? A Systematic Review

**DOI:** 10.3390/ijms26146934

**Published:** 2025-07-19

**Authors:** Teodor Salmen, Claudia Cobilinschi, Andrei Mihăilescu, Bianca-Margareta Salmen, Gabriela-Claudia Potcovaru, Daniela Opris-Belinski, Narcis Copcă, Simona Caraiola, Florentina Negoi, Anca Pantea Stoian, Ioana Săulescu

**Affiliations:** 1 Doctoral School, “Carol Davila” University of Medicine and Pharmacy, 050474 Bucharest, Romania; teodor.salmen@gmail.com (T.S.); bianca-margareta.mihai@drd.umfcd.ro (B.-M.S.); claudia-gabriela.potcovaru@drd.umfcd.ro (G.-C.P.); flori.negoi28@gmail.com (F.N.); 2 Faculty of Medicine, “Carol Davila” University of Medicine and Pharmacy, 050474 Bucharest, Romania or daniela.opris@umfcd.ro (D.O.-B.); narcis.copca@umfcd.ro (N.C.); simona.caraiola@umfcd.ro (S.C.); anca.stoian@umfcd.ro (A.P.S.); ioana_oprisan@yahoo.com (I.S.); 3 Department of Rheumatology and Internal Medicine, Sfânta Maria Clinical Hospital, 011172 Bucharest, Romania; 4 Department of Surgery II, Sfânta Maria Clinical Hospital, 011172 Bucharest, Romania; 5 Department of Rheumatology and Internal Medicine, Colentina Clinical Hospital, 072202 Bucharest, Romania; 6 Department of Diabetes, Nutrition and Metabolic Diseases, “Carol Davila” University of Medicine and Pharmacy, 020021 Bucharest, Romania

**Keywords:** advanced glycation end products, skin autofluorescence, biomarker, systemic lupus erythematosus

## Abstract

Systemic lupus erythematosus (SLE) is a multifaceted autoimmune disease with a heterogeneous organ involvement, for which reliable biomarkers are still being studied. The implication of advanced glycation end products (AGEs), resulting from oxidative stress, and their interaction with the receptor for AGEs (RAGE) has been studied in pathologies with chronic proinflammatory status, offering potential relevance in SLE. This systematic review aimed to evaluate the utility of skin autofluorescence (SAF)—a non-invasive proxy for AGE accumulation—as a biomarker for disease severity, activity, and impact in SLE patients. Following PRISMA guidelines, six studies assessing SAF and/or circulating AGEs and soluble RAGE (sRAGE) in SLE were analyzed. Findings consistently showed higher AGE levels in SLE patients compared to healthy controls, with several correlations between SAF/AGEs and disease features such as SLEDAI scores, organ involvement, inflammatory markers, and damage indices. Decreased sRAGE levels were also observed, possibly due to consumption by AGEs. Some studies further reported predictive associations between specific AGEs or their ratios with sRAGE and particular clinical phenotypes. Although heterogeneity among studies limits definitive conclusions, the AGEs–sRAGE axis—and especially SAF—emerges as a promising candidate for future biomarker development in SLE. Further large-scale longitudinal studies are needed to confirm its clinical utility.

## 1. Introduction

Systemic lupus erythematosus (SLE) is an autoimmune disease of unclear causes, one of the several illnesses known as the great imitator, in which the immune system may mistakenly attack healthy tissue in multiple organs or areas of the body, mainly through the production of autoantibodies [1]. Symptoms may vary among people and can range from mild to severe, in an evolving manner, often with intricate periods of flare and remission [2]. Treatment for the ailment may include a vast array of drugs, among which corticosteroids, immunosuppressants, and immunomodulators are counted, while the long-term prognosis is often variable [3].

SLE pathogenesis is intricate and still unclear, but it is known that environmental factors (UV radiations, airborne pollutants, smoking, drugs), genetic factors (polymorphisms and mutations in the genes of major histocompatibility complex, complement system, nucleic acids metabolism), hormonal factors (imbalance of the female reproductive hormones) and impaired clearance of dying cells are involved in the development of the disease [4,5]. More recently, chronic inflammation in SLE was linked to an intensified glycation process [6].

Advanced glycation end products (AGEs) comprise a class of compounds found in body fluids, cells, and tissues, resulting from the glycation of proteins, lipids, or nucleic acids under conditions of oxidative stress and hyperglycemia [7]. There is a panoply of AGEs, amongst which carboxymethyllysine, carboxyethyllysine, pentosidine, and fructosamine are the most acknowledged, with many more currently being studied [8]. Their receptor (RAGE), belonging to the immunoglobulin superfamily, is expressed by numerous types of immune cells (including T-lymphocytes, macrophages, and neutrophils) [9,10].

AGEs are known to accumulate in certain diseases involving a chronic state of inflammation (such as cardiovascular (CV) disease, diabetes mellitus (DM), chronic renal failure, Alzheimer’s and more recently rheumatic illnesses like rheumatoid arthritis (RA) or SLE), stimulating the AGE receptors (RAGEs) and therefore causing an amplification of the already present inflammation through production of type 1 interferon, tumor necrosis factor α, and/or interleukin 6 via intracellular activation of the NF-KB pathway [11,12,13,14]. The amassment of AGEs also directly leads to structural alterations and gradual dysfunction of cells and tissues [6].

RAGE has a soluble variant (sRAGE), composed of only the extracellular ligand-binding domain, which has the same ligand-binding specificity as its membrane counterpart and hence acts as a decoy for the AGEs by preventing their connection to the cell surface receptor. sRAGE can also directly bind to RAGE [13,14,15,16].

The recently discovered AGEs–sRAGE axis seems to play an important role in different disorders which have inflammation at their core, including SLE. As such, gathering adequate information about it and its possible associations with specific illness characteristics might empower its components to serve as valuable biomarkers in SLE, with the aim to improve prognosis and to prevent irreversible damage. Evaluation of tissue accumulation of AGEs might represent a useful tool for measuring the impact of this process, and the skin could be within easy reach. From this point of view, skin autofluorescence (SAF) can evaluate AGE deposition, providing important information about the impact of an otherwise silent phenomenon in SLE—chronic inflammation [6,7,10].

Identifying different markers to help us anticipate the severity or the potential of evolution and, even more, the impact of the disease on the patients suffering from SLE is a very important element that will help us in stepping closer to personalized medicine [17]. The heterogeneity of affected patients’ types, of the form of disease manifestation, and of the environmental factors that cause SLE means the identification of a predictive marker is of the utmost importance in a medical clinic setting. Because there are no clear guidelines for predicting SLE evolution, this systematic review aims to determine the utility of SAF as a potential marker in predicting SLE severity, evolution, and impact of the disease in patients with SLE.

## 2. Materials and Methods

A systematic review was performed according to the guidelines and recommendations from the Preferred Reporting Items for Systematic Reviews and Meta-Analysis Checklist (PRISMA). The protocol for this review has been registered with the identifier CRD42024536183.

### 2.1. Research Question and Search Strategy

An electronic search for relevant publications was performed using the PubMed, Scopus, and Web of Science library databases from 1 January 2014 to 2025. The following search strategy was used: “skin autofluorescence AND systematic lupus erythematosus”. In this search, 257 articles were found (70 from PubMed, 100 from Scopus, and 87 from Web of Science). After applying filters for language (English), publication type (original articles), and date range (2014 to the date of the search), 72 articles remained. We eliminated 7 duplicates, and then the remaining 65 articles underwent initial title screening, followed by abstract review by two independent reviewers. After excluding studies focused on populations other than the one of interest, those with outcomes different from the ones of interest, as well as meta-analyses and study protocols, 4 articles remained for full assessment. Afterward, 2 more studies were identified by manual search of the reference list of the relevant articles and were added.

The research question was framed using the Population, Intervention, Comparison, and Outcome (PICO) method. The population was represented by patients with SLE and assessed SAF levels; intervention was represented by the SAF levels and their evaluated relationship with SLE evolution, severity, or impact on the patients with SLE. The outcome was defined by the number and/or presence and/or grade of the identified parameters in SLE patients as compared to healthy individuals, with effect measured by percentage, confidence interval (CIs), odds, or relative risks.

### 2.2. Inclusion Criteria

To be included in this review, studies had to meet the following publication criteria: (i) original full-text articles with cohort and cross-sectional studies; (ii) articles from the last ten years; (iii) articles published in English; (iv) conducted on adult human populations.

### 2.3. Exclusion Criteria

Studies were excluded from the analysis if they (i) were on patients without SAF levels measured, (ii) lacked the SLE diagnosis, (iii) had comments, letters to editors, or reviews, or (iv) were on cell cultures or on mammals.

### 2.4. Selection of Studies

Studies that met the following eligibility criteria were included: (1) the study included patients with SLE in whom SAF was assessed; (2) SAF level and its relationship with number, presence, or severity of SLE activity were reported; and (3) the study provided sufficient information such as the corresponding 95% CIs or at least *p*-value. Studies were excluded if they met any of the following: (1) if they were a letter to the editor, expert opinion, case report, meeting abstract, or review; (2) if they were a redundant publication; or (3) if they needed more precise or complete data.

Because the initial selection resulted in only 4 studies, which is very few, and because the references search provided the possibility to enlarge the number to 6, with valuable studies, even if they were older than 10 years, we added them for a better presentation of the subject of interest.

### 2.5. Data Extraction

Two authors used a self-made data extraction table to individually evaluate and extract the following data for each included literature reference: the first author and year of publication, country, study duration, study design, sample size, average age of participants, SAF model, baseline SAF level, and reported parameters and their outcomes as 95% CIs or mean values. Any differences of opinion were settled through discussion or consultation with a third author.

### 2.6. Risk of Bias Assessment

Two reviewers independently assessed the quality of the studies using the Newcastle–Ottawa Scale (NOS), a star rating system that evaluates articles on selection, comparability, and outcome criteria [18]. Research papers rated with at least six stars as evaluated on the NOS scale are considered of good quality and included in the Results Section. This selection process is summarized in Table 1.

### 2.7. Strategy for Data Synthesis

A narrative synthesis of the findings in the studies is centered around the SAF level and its relationship with SLE evolution, severity, progression and impact, and outcomes. The studies are anticipated to be heterogeneous (study design, study quality, screening methods described, interventions, and outcomes). Therefore, it is expected that a narrative synthesis will be performed using text and tables to provide a descriptive summary and explanation of study characteristics and findings.

## 3. Results

**Figure 1:**
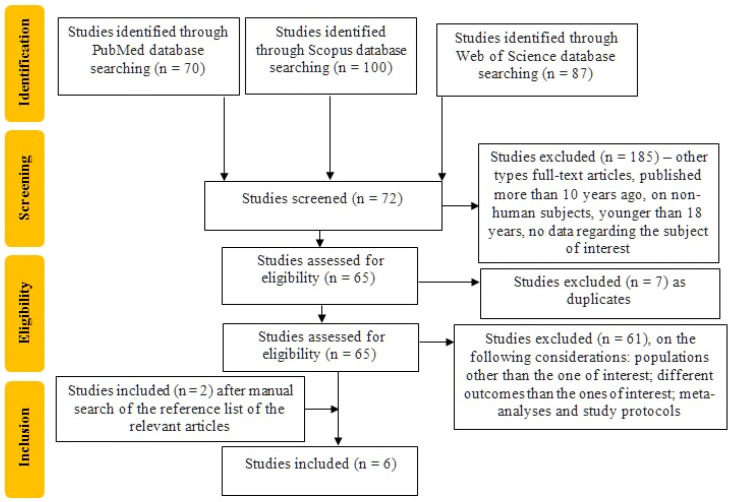
Flowchart on the study selection.

This systematic review incorporates six studies published between 2007 and 2024, as seen in Figure 1.

Characteristics of the included studies, such as author, country, study period and design, sample size, mean age of the included patients, and SAF model, are shown in Table 2.

The main results of the included studies are shown in Table 3.

## 4. Discussion

SLE complexity, from both a pathogenic and a clinical point of view, explains the paucity of well-established biomarkers required for better understanding and tracking disease evolution and treatment response [24].

A 2007 Dutch study evaluated the concentration of AGEs in tissues by measuring skin autofluorescence (SAF) in 55 SLE patients (excluding those with an active disease within the last 4 months, pregnant women, and those with DM) and in an equal amount of age- and gender-matched healthy controls (HCs) in order to make a comparison. Total CV risk, calculated using SCORE, did not differ between the two groups [23].

- It turned out AGE levels were higher in SLE patients as opposed to HCs.
- The study also indicated AGE accumulation might contribute to accelerated atherosclerosis in SLE while being moderately correlated with intima media thickness but also with patient age, creatinine levels, affliction’s duration (even after correction for age), and damage index.
- No association was found between SAF and immunosuppressive treatment.

Another Dutch study from 2008 strived to estimate tissue deposition of AGEs by measuring SAF but also calculated plasma levels of particular AGEs (carboxymethyllysine and carboxyethyllysine) through tandem mass spectrometry and serum levels of sRAGE with the help of ELISA kits. A total of 10 SLE patients (all evaluated during both active and inactive illness, according to the disease activity index) and 10 age- and sex-matched HCs were introduced in the study, comparing them head-to-head. Traditional CV risk factors were similar between the two groups. Pregnant women, diabetics, and patients with renal failure were excluded [22].

- Skin AGE levels and, surprisingly, blood sRAGE levels were significantly higher in SLE patients (more so if the disease was in an active state), whereas blood levels of the specific AGEs were comparable to those of HCs.
- Blood sRAGE levels were positively correlated with anti-dsDNA antibody levels and negatively correlated with age, systolic blood pressure values, and C4 levels.
- No correlation was found between skin AGE levels and CRP values or disease duration.

A 2012 Chinese study assessed the plasma levels of sRAGE using ELISA kits for more than 100 SLE patients and for a certain number of HC, confronting the two groups [21].

- Average sRAGE level in patients with SLE (no matter if active or inactive, treated or untreated; indifferent to patient age and disease duration) was significantly lower than for HCs.
- Interestingly, there seems to be a noticeable difference for average sRAGE level between patients who started treatment less than 1 month before the study and those who had already been receiving it for more than 1 month; the first group had significantly lower levels even to the untreated patients, while the second group had comparable levels to HCs.
- The study also found significantly increased sRAGE plasma levels for patients with rash and serositis compared to those who did not present these clinical features.
- For other evaluated SLE clinical traits (e.g., arthritis, myositis, nephritis, vasculitis), no relevant distinctions were noticed.
- sRAGE plasma levels were not correlated with autoantibodies production (ANA, anti-dsDNA, anti-Sm).

A 2021 Polish study aimed to ascertain the concentrations of total AGEs, particular ones (carboxymethyllysine, carboxyethyllysine, and pentosidine), and sRAGE simultaneously using ELISA kits for the serum samples of more than 30 women suffering from SLE and of an equal amount of HCs, contrasting the two groups. Diabetic patients were excluded altogether [6].

- Concentrations of total AGEs were significantly higher in SLE patients compared to HCs, with none of the specifically assessed compounds (carboxymethyllysine, carboxyethyllysine, and pentosidine) following the same pattern.
- More so, a decrease in sRAGE concentration was observed in the serum samples of SLE patients compared to HCs.

Another Polish study from 2023 determined the concentration of AGEs in tissues through the use of SAF in patients suffering from different rheumatic diseases, including almost 30 with SLE. Depression was the main separator for the study groups, while CV disease (CVD) (high blood pressure, obesity, dyslipidemia, and atherosclerosis), DM, and smoking were present in matching proportions for all groups to preserve the practical value of this research [20].

- The results indicated that the presence of depression may influence the increase in AGE concentration in the skin of SLE patients.

A 2024 Spanish study tried to elucidate the role of skin AGEs in more than 60 SLE patients by measuring through SAF their concentrations and comparing them to age- and sex-matched HCs having at least one CV risk factor present while also searching for correlations between AGE concentrations and SLE characteristics [25].

- It turned out that skin AGE levels were significantly higher for SLE patients than for HCs.
- Skin AGE concentrations positively correlated with a higher frequency of oral ulcers and leukocyturia, a higher level of inflammation biomarkers (CRP, IL6), a higher level of C3 and C4, a higher disease activity index (SLEDAI), and a higher disease damage index (SDI).
- Skin AGE concentrations negatively correlated with autoantibodies production (ANA, anti-Ro60).

Another 2024 Spanish study evaluated the tissue concentrations of AGEs by measuring SAF, determined the serum levels of specific AGEs (carboxymethyllysine, carboxyethyllysine, and pentosidine) and of sRAGE with the help of ELISA kits, and calculated the ratios between AGEs and sRAGE. The objective was to establish a potential link between these parameters and various disease characteristics for approximately 120 SLE patients. Exclusion criteria were pregnancy, DM, active malignancy, fibromyalgia, and treatment with a dose equivalent of prednisone > 20 mg/day [26].

- Multiple positive correlations were discovered:
- Pentosidine with pulmonary manifestations (lupus pneumonitis and shrinking lung syndrome);
- Carboxymethyllysine with non-Caucasian ethnicities, anti-dsDNA antibodies, IL6, and disease duration;
- Carboxyethyllysine with anti-dsDNA antibodies, IL6, and number of accumulated manifestations throughout the illness;
- sRAGE with female gender, photosensitivity, and specific treatments (belimumab, rituximab, and mycophenolic acid);
- Pentosidine/sRAGE ratio with anti-Ro52 antibodies;
- Carboxymethyllysine/sRAGE ratio with non-Caucasian ethnicities, densitometric osteoporosis, and SDI;
- Carboxyethyllysine/sRAGE ratio with CRP and IL6.
- Negative correlations were discovered as well:
  ○ sRAGE with male gender;
  ○ Pentosidine/sRAGE ratio with biological treatment;
  ○ Skin AGEs/sRAGE ratio with female gender and disease duration.
- Amazingly, no correlation was observed between the studied parameters and CV risk factors or events.

The overall concentration of AGEs in the blood and skin is significantly higher in SLE patients; however, it is currently impossible to pinpoint which specific compounds are increased [27]. Progress in the research of distinctive AGEs and the pathways leading to their formation will help us to better understand this phenomenon and to potentially find markers of glycation useful in diagnosing SLE [28].

The association of particular blood AGEs with specific SLE clinical manifestations and biological alterations (e.g., pentosidine with pulmonary manifestations, carboxymethyllysine and carboxyethyllysine with anti-dsDNA antibodies and IL6) may eventually outline certain phenotypes of the disease, with the respective AGEs representing a strong predictor for the illness course [20,29].

Decreased sRAGE serum levels in SLE patients are most likely explained through its secondary consumption by excessively generated AGEs, but a primary deficit might also be involved in some cases. Furthermore, transiently decreased sRAGE levels also appear to occur due to the initiation of treatment, while the prolonged use of specific immunosuppressants (belimumab, rituximab, and mycophenolic acid) seems to be associated with an increase in sRAGE levels. The investigation of this soluble receptor for SLE patients needs to expand in order to reach detailed findings, since discrepant results are currently available [30].

Ratios between specific serum AGEs or total skin AGEs and sRAGE might also prove to be highly suitable biomarkers in SLE, but more exploration of the matter needs to be completed [31].

All in all, the AGEs–sRAGE axis presents potential for having its place and applications in the future routine clinical practice of SLE, improving the diagnosis, management, and/or prognosis of the ailment as well as of multiple comorbidities.

The latest data shows that SLE patients with non-threatening organ involvement (e.g., skin and joints) have more damage accrual over time because they are less stringently monitored and treated [26]. As such, new EULAR recommendations for SLE treatment emphasize the importance of having remission or at least low disease activity as a target. Still, in clinical practice, there is a tendency to use lighter treatment for SLE forms with only mild to moderate activity, where clinicians are not aware of subclinical inflammation and subsequent damage [4,32,33].

Our review’s main limitation is the small number of studies included, alongside the diversity of the reported data pertaining to various clinical, biological, and therapeutic aspects of patients with SLE, without evaluating or taking into account comorbidities such as DM or chronic kidney disease. Conversely, the strength of the present paper is based on its effort to provide the overall picture of what is known about SLE and SAF.

## 5. Conclusions

AGE concentration assessment in SLE patients also appears to be useful for establishing those who are at risk of developing more severe forms of the ailment and comorbidities such as CVD, DM, renal failure, and depression (which can emerge due to progression of the illness or prolonged exposure to certain medications). As such, this evaluation might prove a valuable tool for guiding further investigations, calibrating treatment, and monitoring disease activity and/or damage.

Long-term, large-scale, and prospective studies are needed to appraise if modulation of AGEs and/or sRAGE levels can be considered a viable treatment option to prevent or at least delay organ damage in SLE.

## Figures and Tables

**Table 1:** Newcastle–Ottawa Scale analysis of the included articles [6,19,20,21,22,23].

Author (Reference)	Selection				Comparability	Outcome			Total Score	Quality
	Representativeness of the Exposed Cohort	Selection of the Non-Exposed Cohort	Ascertainment of Exposure	Demonstration that Outcome of Interest Was Not Present at Start of Study	Comparability of Cohorts Based on the Design or Analysis	Assessment of Outcome	Was Follow-Up Long Enough for Outcomes to Occur	Adequacy of Follow-Up of Cohorts		
Carrión-Barbera 2024 [19]	+	+	+	+	-	+	+	+	7	Good
Żuchowski 2023 [20]	+	+	+	+	-	+	+	+	7	Good
Nowak 2021 [6]	+	+	+	+	-	+	+	+	7	Good
Ma 2012 [21]	+	-	+	+	-	+	+	+	6	good
Nienhuis 2008 [22]	+	-	+	+	-	+	+	+	6	good
de Leeuw 2007 [23]	+	+	+	+	-	+	+	+	7	good

“+” indicates that the article meets the criteria mentioned above; “-” indicates that the article does not meet the abovementioned criteria.

**Table 2:** Characteristics of the included studies.

Author	Country	Study Period	Study Design	Sample Size	Mean Age	SAF Model
Carrión-Barberà I. et al. [19], 2024	Spain	NR	Cross-sectional study	251	50.4 ± 14.9	AGE Reader Mu ConnectR, DiagnOptics Technologies BV, Groningen, The Netherlands
Żuchowski P. et al. [20], 2023	Poland	2 years	Case-control study	139	45.3 ± 16.1	AGE Reader device (DiagnOptics BV, Groningen, The Netherlands)
Nowak A. et al. [6], 2021	Poland	1 year	Case-control study	57	56.39 ± 11.36	OxiSelectTM AGE Competitive Elisa Kit (Cell Biolabs, Inc., San Diego, CA, USA)
						RayBio^®^ Human RAGE ELISA Kit, catalogue number ELH-RAGE (RayBiotech, Norcross, GA, USA)
Ma C.Y. et al. [21], 2012	China	NR	Cross-sectional study	148	32.4 ± 11.3	ELISA kit (R&D systems, Minneapolis, MN, USA)
Nienhuis H.L. et al. [22], 2008	Holland	NR	Cross-sectional study	20	29 (22, 39)	AF-EEMS, using UV-A radiation
de Leeuw K. et al. [23], 2007	Holland	NR	Cross-sectional study	110	43 ± 12	AF-EEMS

NR—not reported; AGE—advanced glycation end product; SAF—skin autofluorescence; AF-EEMS—Autofluorescence Excitation–Emission Matrix Scanner; ELISA—enzyme-linked immunosorbent assay; UVA—ultraviolet A radiation.

**Table 3:** The main results of the included studies.

Author	Baseline SAF Level	Parameters	Outcome of the Parameter	*p* Value
Carrión-Barberà I. et al. [19], 2024	2.71 ± 0.56 AU	Oral ulcers present	2.65, 95% CI [2.5, 2.8]	*p* = 0.0304
		Oral ulcers absent	2.44, 95% CI [2.31, 2.56]	NR
		PtGA > 3	2.69, 95% CI [2.54, 2.85]	*p* = 0.01
		PtGA [0, 3]	2.43, 95% CI [2.31, 2.55]	NR
		PGA > 2	2.52, 95% CI [2.34, 2.7]	*p* = 0.0104
		PGA [1, 2]	2.46, 95% CI [2.32, 2.6]	*p* = 0.0181
		PGA = 0	2.13, 95% CI [1.87, 2.38]	NR
		SDI [5, 6]	3.03, 95% CI [2.45, 3.61]	*p* = 0.0156
		SDI [3, 4]	2.25, 95% CI [1.87, 2.62]	*p* = 0.714
		SDI [0, 2]	2.32, 95% CI [2.2, 2.44]	NR
		SLEDAI severe	2.85, 95% CI [2.52, 3.18]	*p* = 0.0032
		SLEDAI moderate	2.53, 95% CI [2.37, 2.7]	*p* = 0.0493
		SLEDAI remission—mild	2.33, 95% CI [2.2, 2.46]	NR
		IL-6 [3.33, 144.1]	2.58, 95% CI [2.39, 2.76]	*p* = 0.0068
		IL-6 [1.88, 3.33)	2.42, 95% CI [2.23, 2.61]	*p* = 0.1197
		IL-6 [0.63, 3.33)	2.22, 95% CI [2.03, 2.41]	NR
		C4 [24, 49]	2.54, 95% CI [2.37, 2.71]	*p* = 0.015
		C4 [18, 24)	2.51, 95% CI [2.34, 2.68]	*p* = 0.0335
		C4 [2, 18)	2.26, 95% CI [2.09, 2.43]	NR
		Anti-nuclear present	2.49, 95% CI [2.37, 2.61]	*p* = 0.028
		Anti-nuclear absent	2.99, 95% CI [2.56, 3.41]	NR
		Anti-Ro60 present	2.38, 95% CI [2.18, 2.57]	*p* = 0.0359
		Anti-Ro60 absent	2.64, 95% CI [2.49, 2.79]	NR
		Leukocyturia [2, 5]	2.7, 95% CI [2.48, 2.93]	*p* = 0.0052
		Leukocyturia = 1	2.48, 95% CI [2.26, 2.7]	*p* = 0.2342
		Leukocyturia = 0	2.33, 95% CI [2.2, 2.47]	NR
		CRP [0.28, 3.92]	2.58, 95% CI [2.41, 2.75]	*p* = 0.0233
		CRP [0.12, 0.28)	2.41, 95% CI [2.23, 2.59]	*p* = 0.4272
		CRP [0.03, 0.12)	2.32, 95% CI [2.16, 2.48]	NR
Żuchowski P. et al. [20], 2023	2.2 ± 0.5 AU	AGE in depression versus without depression groups	2.5 ± 0.4 versus 2 ± 0.5	*p* = 0.024
Nowak A. et al. [6], 2021	AGE = 30.51 ± 6.80 μg/mL	AGE in SLE group versus control group	30.51 ± 6.80 versus 24.02 ± 8.50	*p* < 0.01
	sRAGE = 47.18 ± 19.41 Pg/mL	sRAGE in SLE group versus control group	36.36 ± 15.71 versus 47.18 ± 19.41	*p* < 0.05
Ma C.Y. et al. [21], 2012	RAGE 842.7 ± 50.6 Pg/mL	sRAGE levels in SLE versus healthy groups	842.7 ± 50.6 Pg/mL versus 1129.3 ± 80.1 Pg/mL	*p* = 0.003
		sRAGE levels in inactive SLE versus healthy controls	761.7 ± 77.2 Pg/mL versus 1129.3 ± 80.1 Pg/mL	*p* = 0.003
		sRAGE levels in active SLE versus healthy controls	876.6 ± 63.9 Pg/mL versus 1129.3 ± 80.1 Pg/mL	*p* = 0.012
		sRAGE levels in inactive SLE versus active SLE patients	761.7 ± 77.2 Pg/mL versus 876.6 ± 63.9 Pg/mL	*p* = 0.303
		sRAGE levels in untreated SLE patients versus healthy controls	865.0 ± 81.5 Pg/mL versus 1129.3 ± 80.1 Pg/mL	*p* = 0.035
		sRAGE levels in treated SLE patients versus healthy controls	833.8 ± 63.1 Pg/mL versus 1129.3 ± 80.1 Pg/mL	*p* = 0.004
		sRAGE levels in untreated SLE patients versus treated SLE patients	865.0 ± 81.5 Pg/mL versus 833.8 ± 63.1 Pg/mL	*p* = 0.782
		sRAGE in SLE patients with rash versus patients without rash	973.4 ± 91.0 Pg/mL versus 759.0 ± 57.2 Pg/mL	*p* = 0.039
		sRAGE in patients with serositis versus patients without serositis	1201.9 ± 209.1 Pg/ml versus 804.9 ± 50.3 Pg/mL	*p* = 0.02
		sRAGE between patients with normal (>90 mL/min/1.73 m^2^) versus lower eGFR (<90 mL/min/1.73 m^2^)	887.7 ± 82.5 Pg/mL versus 949.5 ± 155.1 Pg/mL	*p* = 0.733
		sRAGE levels in patients with SLE and ANA versus negative ANA	NR	*p* > 0.05
		sRAGE levels in patients with SLE and anti-dsDNA versus negative anti-dsDNA	NR	*p* > 0.05
		sRAGE levels in patients with SLE and AnuA versus negative AnuA	NR	*p* > 0.05
		sRAGE levels in patients with SLE and anti-Sm versus negative anti-Sm	NR	*p* > 0.05
		sRAGE levels and leucocyte count	*n* = 95, r = −0.326,	*p* = 0.001
		sRAGE levels and lymphocytes count	*n* = 95, r = −0.357	*p* < 0.0001
		sRAGE levels and leucocyte count	*n* = 95, r = −0.272	*p* = 0.008
		sRAGE levels and monocyte count	*n* = 95, r = −0.286	*p* = 0.005
Nienhuis H.L. et al. [22], 2008	NR	sRAGE levels in patients with active versus quiescent SLE	3752 Pg/mL (3107–5570) versus 2882 Pg/mL (2363–3539)	*p* < 0.05
		sRAGE levels in patients with quiescent SLE versus control	2882 Pg/mL (2363–3539) versus 2107 Pg/mL (1771–2538)	*p* < 0.05
		AF-EEMS levels in patients with quiescent SLE versus control	NR versus 1.09 AU (0.77–1.50)	*p* < 0.05
de Leeuw K. et al. [23], 2007	NR	AF-EEMS levels in patients with SLE versus control	1.50 ± 0.5 AU versus 1.28 ± 0.4 AU	*p* = 0.006
		AF-EEMS levels in smokers versus non-smokers	1.42 ± 0.4 AU versus 1.38 ± 0.5 AU	*p* > 0.05
		AF-EEMS levels in males verus females	1.55 ± 0.7 AU versus 1.36 ± 0.4 AU	*p* > 0.05
		AF-EEMS levels in manifest CVD versus non-manifest CVD	1.54 ± 0.4 AU versus 1.49 ± 0.6 AU	*p* > 0.05
		AF-EEMS levels in patients with prednisolone versus patients without	1.48 ± 0.5 AU versus 1.52 ± 0.5 AU	*p* > 0.05
		AF-EEMS levels in patients with antiphospholipid antibodies versus negative antiphospholipid antibodies	1.48 ± 0.4 AU versus 1.51 ± 0.6 AU	*p* > 0.05

NR—not reported; CI—confidence interval; AU—arbitrary units; AGE—advanced glycation end product; sRAGE—soluble receptor for advanced glycation end products; SAF—skin autofluorescence; AF-EEMS—Autofluorescence Excitation–Emission Matrix Scanner; ELISA—enzyme-linked immunosorbent assay; SLE—systemic lupus erythematosus; SLEDAI—SLE disease activity index; SDI–SLE damage index; PGA—physician global assessment; PtGA—patient global assessment; ANA—anti-nuclear antibodies; AnuA—anti-nucleosome antibodies; anti-dsDNA—anti-double stranded deoxyribonucleic acid antibodies; anti-Sm—anti-Smith antibodies; anti-Ro60—anti-protein Ro60 antibodies; CRP—C reactive protein; C4—complement 4 component; IL-6—interleukin 6; CVD—cardiovascular disease; eGFR—estimated glomerular filtration rate; UVA—ultraviolet A radiation.

## Abbreviations

AF-EEMS	Autofluorescence Excitation–Emission Matrix Scanner
AGE	Advanced glycation end product
ANA	Antinuclear antibody
AnuA	Anti-nucleosome antibody
Anti-dsDNA	Anti-double stranded DNA antibody
Anti-Ro60	Anti-protein Ro60 antibody
Anti-Sm	Anti-Smith antibody
AU	Arbitrary units
C4	Complement 4
CI	Confidence interval
CRP	C reactive protein
CV	Cardiovascular
CVD	Cardiovascular disease
DM	Diabetes mellitus
eGFR	Estimated glomerular filtration rate
ELISA	Enzyme-linked immunosorbent assay
HC	Healthy control
IL-6	Interleukin 6
NR	Not reported
PGA	Physician global assessment
PtGA	Patient global assessment
RA	Rheumatic arthritis
SAF	Skin autofluorescence
SDI	SLE damage index
SLE	Systemic lupus erythematosus
SLEDAI	SLE disease activity index
sRAGE	Soluble receptor for advanced glycation end products
UVA	Ultraviolet-A radiation
